# Supplementary material for: Saccharomyces boulardii (CNCM I-745) improves intestinal damage in sepsis by remodeling intestinal flora structure
Source: Front Cell Infect Microbiol. 2025 Jul 31;15:1602792. doi: 10.3389/fcimb.2025.1602792 (PMC12350329; doi:10.3389/fcimb.2025.1602792)
Supplement: Supplementary file 1 [file DataSheet1.zip › summary/summary/8_Advanced_analyse/2_PICRUSt2_result/KEGG/Group.DW4_vs_DW0/PICRUSt2_KO_Group.DW4_vs_DW0_diff.pdf]

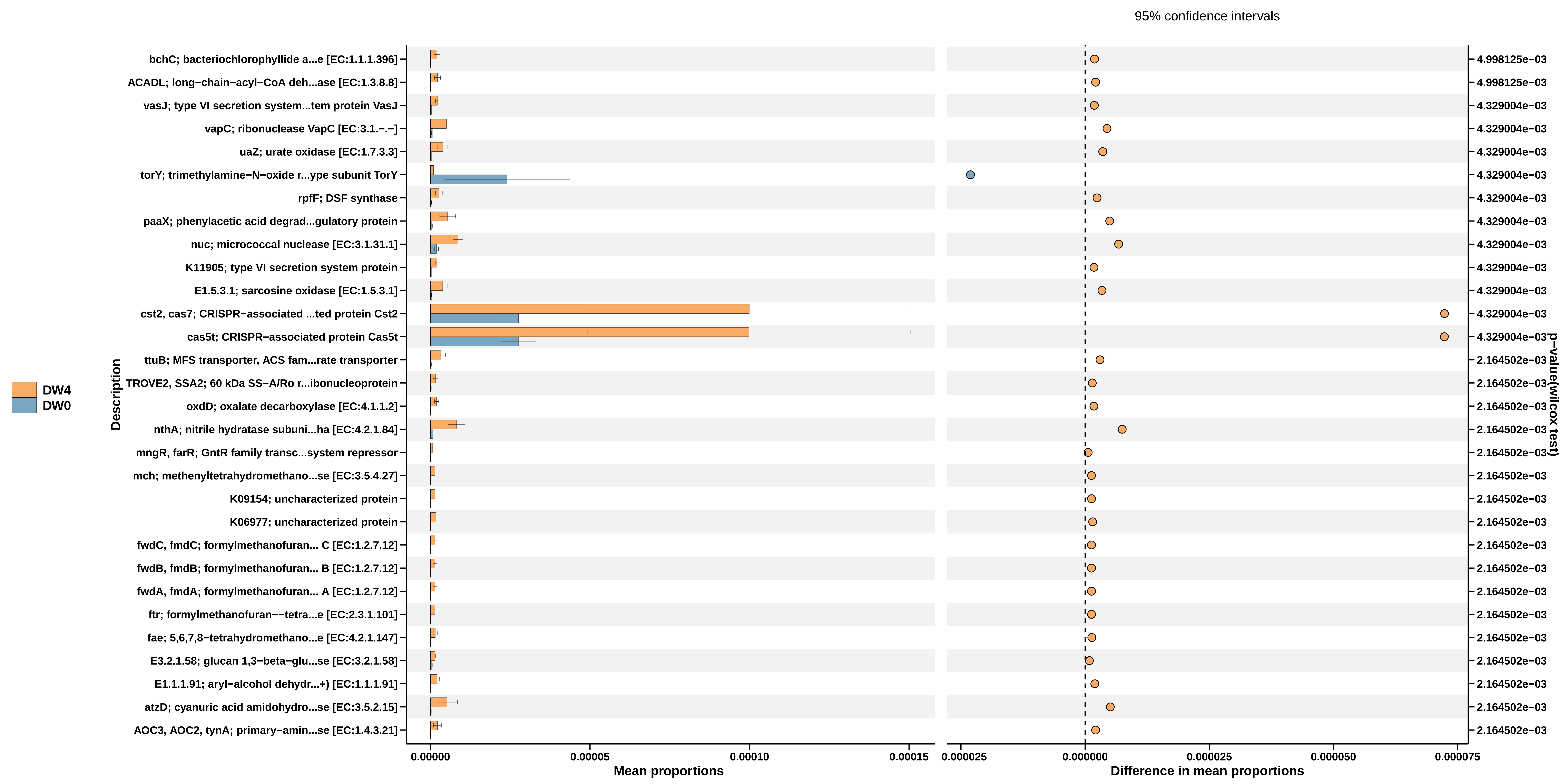

95% confidence intervals
DW4
DW0
Description
bchC; bacteriochlorophyllide a...e [EC:1.1.1.396]
ACADL; long−chain−acyl−CoA deh...ase [EC:1.3.8.8]
vasJ; type VI secretion system...tem protein VasJ
vapC; ribonuclease VapC [EC:3.1.−.−]
uaZ; urate oxidase [EC:1.7.3.3]
torY; trimethylamine−N−oxide r...ype subunit TorY
rpfF; DSF synthase
paaX; phenylacetic acid degrad...gulatory protein
nuc; micrococcal nuclease [EC:3.1.31.1]
K11905; type VI secretion system protein
E1.5.3.1; sarcosine oxidase [EC:1.5.3.1]
cst2, cas7; CRISPR−associated ...ted protein Cst2
cas5t; CRISPR−associated protein Cas5t
ttuB; MFS transporter, ACS fam...rate transporter
TROVE2, SSA2; 60 kDa SS−A/Ro r...ibonucleoprotein
oxdD; oxalate decarboxylase [EC:4.1.1.2]
nthA; nitrile hydratase subuni...ha [EC:4.2.1.84]
mngR, farR; GntR family transc...system repressor
mch; methenyltetrahydromethano...se [EC:3.5.4.27]
K09154; uncharacterized protein
K06977; uncharacterized protein
fwdC, fmdC; formylmethanofuran... C [EC:1.2.7.12]
fwdB, fmdB; formylmethanofuran... B [EC:1.2.7.12]
fwdA, fmdA; formylmethanofuran... A [EC:1.2.7.12]
ftr; formylmethanofuran−−tetra...e [EC:2.3.1.101]
fae; 5,6,7,8−tetrahydromethano...e [EC:4.2.1.147]
E3.2.1.58; glucan 1,3−beta−glu...se [EC:3.2.1.58]
E1.1.1.91; aryl−alcohol dehydr...+) [EC:1.1.1.91]
atzD; cyanuric acid amidohydro...se [EC:3.5.2.15]
AOC3, AOC2, tynA; primary−amin...se [EC:1.4.3.21]
0.00000
0.00005
0.00010
0.00015
Mean proportions
0.000025
0.000000
0.000025
0.000050
0.000075
Difference in mean proportions
p−value(wilcox test)
4.998125e−03
4.998125e−03
4.329004e−03
4.329004e−03
4.329004e−03
4.329004e−03
4.329004e−03
4.329004e−03
4.329004e−03
4.329004e−03
4.329004e−03
4.329004e−03
4.329004e−03
2.164502e−03
2.164502e−03
2.164502e−03
2.164502e−03
2.164502e−03
2.164502e−03
2.164502e−03
2.164502e−03
2.164502e−03
2.164502e−03
2.164502e−03
2.164502e−03
2.164502e−03
2.164502e−03
2.164502e−03
2.164502e−03
2.164502e−03
